# Platelet-Related Biomarkers and Efficacy of Antiplatelet Therapy in Patients with Aortic Stenosis and Coronary Artery Disease

**DOI:** 10.3390/ijms26157083

**Published:** 2025-07-23

**Authors:** Paweł Bańka, Kinga Czepczor, Maciej Podolski, Agnieszka Kosowska, Wojciech Garczorz, Tomasz Francuz, Maciej Wybraniec, Katarzyna Mizia-Stec

**Affiliations:** 1 First Department of Cardiology, School of Medicine in Katowice, Medical University of Silesia, 40-635 Katowice, Poland; 2 Centre of the European Reference Network for Rare, Low Prevalence or Complex Diseases of the Heart (ERN GUARD Heart), 40-635 Katowice, Poland; 3 Department of Biochemistry, Faculty of Medical Sciences in Katowice, Medical University of Silesia, 40-752 Katowice, Poland

**Keywords:** aortic stenosis, platelets, biomarkers, thrombomodulin, platelet factor 4, P-selectin, CD40L, antiplatelet therapy, aspirin

## Abstract

The objective of this study was to evaluate the serum biomarkers implicated in the interaction of platelets and endothelium, as well as the efficacy of antiplatelet therapy in patients with aortic stenosis (AS) and coronary artery disease (CAD). A total of 78 adult patients with CAD on aspirin therapy participated in this study, including 49 consecutive patients with AS and 29 control subjects. The analysis included the following serum biomarkers: thrombomodulin (TM), platelet factor 4 (PF4), P-selectin, and CD40L. The efficacy of antiplatelet treatment was evaluated using the VerifyNow Aspirin test (ASPI test) and P2Y12 assay test (ADP test). Patients with AS exhibited increased serum levels of TM (7.64 ± 3.5 ng/mL vs. 6.28 ± 2.1 ng/mL, *p* = 0.011) and PF4 (25.16; Q1: 8.3; Q3: 29.6 μg/mL vs. 12.85; Q1: 5.7; Q3: 14.5 μg/mL, *p* = 0.021) compared to the control group. P-selectin and CD40L levels did not differ between groups. There were no significant differences in platelet aggregation in the ASPI (474.04 ± 66.7 ARU vs. 471.31 ± 56.2 ARU; *p* = 0.822) or ADP (224.88 ± 46.4 PRU vs. 216.62 ± 29.6 PRU; *p* = 0.394) tests. Bleeding incidence did not differ significantly between groups. The coexistence of AS in patients with CAD is associated with elevated levels of the aforementioned biomarkers, which are indicative of endothelial damage and platelet activation. However, the efficacy of antiplatelet treatment was independent of the presence of AS.

## 1. Introduction

Aortic stenosis (AS) is a progressive valvular heart disease that begins with minor fibrocalcific changes in the leaflets and progresses to more pronounced calcification [1]. Aortic valve sclerosis is a progressive condition that involves endothelial damage, lipid accumulation, and inflammation [2,3,4,5,6,7]. As the disease progresses, the valve opening becomes more constricted, resulting in turbulent blood flow that adversely affects hemodynamics. Turbulent blood flow and shear stress have been demonstrated to contribute to endothelial dysfunction and platelet activation [8,9,10,11,12]. Platelet activation leads to the release of pro-inflammatory cytokines and chemokines [1,7]. Antiplatelet therapy, widely used in the management of cardiovascular diseases, aims to prevent platelet activation and aggregation, thereby reducing the risk of thrombosis. Current and emerging therapeutic interventions target various platelet receptors, including COX-1, P2Y12, and integrin receptors. Aspirin, a COX-1 inhibitor, has been demonstrated to reduce platelet prothrombotic activity, while P2Y12 inhibitors have been shown to block platelet activation via the PI3K/AKT pathway [13,14].

The presence of coronary artery disease (CAD) is common among patients with AS, and antiplatelet therapy is used to prevent cardiovascular incidents in this group of patients. However, AS is characterized by excessive platelet activation, which may influence the efficacy of antiplatelet therapy [15]. Conversely, excessive shear stress exerted on the aortic wall can result in significant degradation of high-molecular-weight multimers of von Willebrand factor, which has been associated with an elevated risk of bleeding, including gastrointestinal bleeding, as observed in Heyde’s syndrome [16,17]. An increasing body of research has evaluated the efficacy and safety of aspirin and P2Y12 receptor inhibitors in the prevention of cardiovascular events among patients with CAD. Several studies have reported a lower risk of bleeding in patients treated with clopidogrel, and clopidogrel is currently used as an alternative to aspirin in various clinical scenarios [18,19,20]. In contrast, data on the effects of AS on platelet function and the safety of antiplatelet therapy in patients with concomitant CAD and AS remain limited and warrant further investigation.

This study evaluates serum biomarkers involved in platelet–endothelium interactions, including thrombomodulin (TM), platelet factor 4 (PF4), and P-selectin, as well as inflammation-related biomarkers such as CD40L.

TM is an integral membrane protein predominantly expressed on the surface of endothelial cells lining blood vessels. It plays a crucial role in the regulation of hemostasis, particularly in the anticoagulant pathway [21]. TM acts as a cofactor for thrombin, a key enzyme in the coagulation cascade, and upon binding, neutralizes thrombin’s procoagulant activity. Impaired TM expression has been observed in damaged or dysfunctional endothelium, contributing to vascular thrombosis [22,23].

PF4 is a small cytokine released from the alpha granules of activated platelets during aggregation. It plays multiple roles in hemostasis, immunity, and inflammation [24]. Complexes of PF4 have been associated with increased platelet activation, inflammation, and an elevated risk of thrombosis [25].

P-selectin, a cell adhesion molecule, is stored in the Weibel–Palade bodies of endothelial cells and the alpha granules of platelets. It plays a pivotal role in the initial rolling of leukocytes, particularly neutrophils and monocytes, along the activated endothelium [26]. P-selectin has also been shown to promote thrombus formation by mediating interactions between platelets, leukocytes, and endothelial cells [27].

CD40L is a transmembrane protein primarily expressed on activated T cells, but it is also found on platelets, B cells, monocytes, and endothelial cells. Activation of CD40 by CD40L stimulates the production of pro-inflammatory cytokines, such as IL-6 and TNF-α, induces the expression of adhesion molecules, and promotes the release of matrix metalloproteinases [28,29]. When expressed on activated platelets, CD40L facilitates the formation of platelet–leukocyte aggregates, endothelial activation, and enhanced thrombus formation [30].

The objective of the present study was to evaluate serum biomarkers associated with platelet–endothelium interactions, including TM, PF4, P-selectin, and CD40L. Additionally, the study aimed to assess the efficacy of antiplatelet therapy in patients with AS and CAD by measuring platelet aggregation.

## 2. Results

### 2.1. Clinical and Laboratory Characteristics

A comparative evaluation of clinical and biochemical characteristics demonstrated statistically significant differences between patients with AS and the control group. Patients with AS exhibited a lower prevalence of tobacco use (14.9% vs. 41.4%, *p* = 0.01). Moreover, these patients were characterized by a higher NYHA class (2.51 ± 0.8 vs. 1.59 ± 1.1, *p* < 0.001). Additionally, patients with AS demonstrated elevated serum creatinine levels (1.13 ± 0.6 mg/dL vs. 0.9 ± 0.1 mg/dL; *p* = 0.012) and reduced estimated glomerular filtration rate (64.91 ± 22.2 mL/min vs. 75.21 ± 15.3 mL/min) compared to the controls. The clinical and the laboratory characteristics are shown in Table 1 and Table 2.

### 2.2. Echocardiographic Characteristics of the AS Group

The echocardiographic characteristics of the study group were as follows: the aortic valve area (AVA) was 0.78 ± 0.2 cm^2^, the maximum transvalvular velocity (Vmax) was 4.28 ± 0.6 m/s, and the mean pressure gradient (Pmean) was 44.82 ± 14.6 mmHg. Among the study group, there were 43 patients with severe AS (23 males, mean age: 75.53 ± 11.65 years) and 6 patients with moderate AS (4 males, mean age: 69.17 ± 16.92 years).

The comparative analysis of echocardiographic parameters between the study groups identified several statistically significant differences, reflecting the underlying pathological characteristics associated with AS. Patients in the AS group demonstrated increased interventricular septum (IVS) thickness (14.49 ± 2.7 mm vs. 11.66 ± 2.9 mm; *p* < 0.001) and posterior wall (PW) thickness (11.29 ± 1.6 mm vs. 9.34 ± 1.5 mm; *p* < 0.001) relative to the control group. A comparative analysis of left ventricular ejection fraction (LVEF) revealed no substantial disparities between the two groups. However, the left ventricular global longitudinal strain (LV GLS) was impaired in the AS group compared to the control group (−13.99 ± 3.1% vs. −16.91 ± 3.3%; *p* = 0.001). The valvulo-arterial impedance (Zva) was notably higher in the AS group (5.5 ± 1.8 vs. 4.47 ± 1.1; *p* = 0.003). Furthermore, the early mitral inflow velocity and the early diastolic mitral annular velocity ratio (E/E′ ratio) were significantly elevated in AS patients (15.88 ± 6.6 vs. 10.43 ± 4.3; *p* < 0.001). The echocardiography characteristics are shown in Table 3.

### 2.3. Platelet Aggregometry

A comparison of the platelet aggregation levels measured using the ASPI test revealed no statistically significant differences between the AS with CAD group and CAD group (474.04 ± 66.7 ARU vs. 471.31 ± 56.2 ARU; *p* = 0.822), nor in the ADP test results (224.88 ± 46.4 PRU vs. 216.62 ± 29.6 PRU; *p* = 0.394).

### 2.4. Biomarkers Serum Levels: AS with CAD vs. CAD

The following differences in biomarkers serum levels were observed between the AS and the control group: a significant increase in TM (7.64 ± 3.5 ng/mL vs. 6.28 ± 2.1 ng/mL, *p* = 0.011) and PF4 (25.16; Q1: 8.3; Q3: 29.6 μg/mL vs. 12.85; Q1: 5.7; Q3: 14.5 μg/mL, *p* = 0.021). In contrast, P-selectin and CD40L levels did not differ significantly between the groups. The results are presented in Table 4.

### 2.5. Correlation Analysis

Pearson’s correlation analysis revealed several statistically significant relationships. A negative correlation was observed between ADP test values (expressed in PRU) and both hemoglobin (r = −0.38; *p* = 0.001) and hematocrit (r = −0.487; *p* = 0.001). Additionally, TM levels showed a negative correlation with hemoglobin (r = −0.298; *p* = 0.008) and hematocrit (r = −0.337; *p* = 0.003). A positive correlation was identified between P-selectin and CD40L concentrations (r = 0.362; *p* = 0.011). No significant correlations were found between the biochemical markers and AS severity. The detailed results are presented in Table 5 and Table S1 in the Supplementary Materials.

### 2.6. Results of 12-Month Follow-Up

After a 12-month follow-up period, the following observations were obtained. The incidence of major bleeding (BARC > 1) did not demonstrate statistically significant differences between the two groups (4.1% vs. 10.3%; *p* = 0.275).

## 3. Discussion

The present study provides new insights into the complex interplay between platelet activation, endothelial dysfunction, inflammation, and efficacy of antiplatelet therapy in patients with AS. We observed significantly elevated levels of TM and PF4 in patients with concomitant AS and CAD compared to those with CAD alone. These findings underscore the heightened endothelial dysfunction and platelet activation associated with AS, suggesting that valvular pathology independently contributes to the hemostatic and inflammatory profile. Our results support the hypothesis that altered hemodynamics in AS, particularly turbulent flow and increased shear stress, contribute to a prothrombotic and proinflammatory microenvironment within the aortic valve [1,7]. Such mechanical stress can activate platelets, leading to the release of PF4 and other mediators implicated in clot formation.

The elevated TM levels observed in AS patients suggest ongoing endothelial injury. TM, an integral membrane protein expressed on endothelial cells, plays a crucial role in the regulation of coagulation and inflammation [31]. Its increased serum levels may reflect a compensatory response to persistent shear stress and endothelial dysfunction. In AS, elevated TM levels likely represent an effort to modulate thrombin activity and prevent excessive clot formation. The significantly higher TM levels in the AS with CAD group suggest that AS exacerbates endothelial stress beyond that observed in CAD alone. This finding is consistent with previous studies showing that AS is associated with mechanical stress-induced endothelial disruption, inflammatory signaling, and extracellular matrix remodeling within the aortic valve [1,12,32,33].

Similarly, PF4, a chemokine stored in platelet alpha granules and released upon activation, serves as a marker of ongoing platelet activation and is implicated in thrombo-inflammatory processes in cardiovascular diseases [24,25]. The higher PF4 levels in patients with AS and CAD suggest that AS contributes to a prothrombotic state, likely driven by altered hemodynamics and increased shear stress across the stenotic valve. This prothrombotic milieu may increase the risk of thromboembolic events [34].

Our findings revealed a selective elevation of TM and PF4 levels in patients with AS and CAD, while P-selectin and CD40L levels remained unchanged. This suggests that AS-related hemodynamic disturbances, particularly increased shear stress and turbulent flow, may predominantly trigger specific endothelial and platelet responses. TM elevation likely reflects chronic endothelial injury and a compensatory attempt to regulate thrombin activity, whereas PF4 indicates primary platelet activation localized to areas of high shear stress. In contrast, P-selectin and CD40L are involved in secondary inflammatory signaling and may exhibit more transient or localized release, which might not be captured in systemic measurements at a single time point. Furthermore, while TM and PF4 elevations are statistically significant, we acknowledge their large interindividual variability, which currently limits their application as reliable individual prognostic markers. Nevertheless, these biomarkers provide valuable mechanistic insight into the interplay between valve pathology, endothelial dysfunction, and platelet activation in AS. Future studies should aim to validate TM and PF4 as potential tools for risk stratification and to explore whether patients with persistently elevated levels may benefit from personalized therapeutic strategies.

Importantly, we did not observe a significant difference in platelet aggregation in response to aspirin therapy between AS patients and controls. Aspirin administration resulted in adequate platelet inhibition regardless of AS presence. The analysis revealed comparable ARU values in both groups, all below 550, indicating effective platelet inhibition. These findings suggest that excessive platelet activation in AS may occur locally at sites of high shear stress on the aortic valve and aortic wall [1]. Despite enhanced platelet activation, functional responsiveness to aspirin appears preserved. Thus, standard antiplatelet therapy may remain effective in reducing platelet aggregation in this cohort, although the potential for aspirin resistance cannot be entirely excluded and warrants further investigation. Baseline platelet function and the degree of platelet inhibition were also assessed using an ADP assay. This assessment is limited, as patients in this study were treated with aspirin alone and did not receive P2Y12 inhibitors. In both groups, PRU values were similar and above 180, indicating inadequate platelet inhibition by ADP pathway blockade, as expected in the absence of P2Y12 inhibitor therapy. AS did not appear to impact this aspect of platelet function assessment.

We also identified a negative correlation between platelet activation, as measured by the ADP test (PRU), and hemoglobin and hematocrit levels. This may be explained by the observation that blood loss and hemolysis can enhance platelet activation [35]. Hemolysis leads to the release of ADP from erythrocytes, which in turn promotes platelet activation and aggregation [35,36,37]. This mechanism may influence the results of ADP-based platelet function tests. Some studies have suggested that lower hemoglobin and hematocrit levels are associated with increased platelet reactivity [38,39,40]. In the presence of hemolysis, ADP assays may falsely indicate heightened platelet aggregation even in the setting of normal platelet function, potentially leading to misinterpretation and inappropriate clinical decisions [41]. Thus, consideration of hemolysis is critical when interpreting ADP test results, particularly in clinical contexts where hemolysis is suspected.

This study also assessed bleeding events (BARC > 1) at 12-month follow-up. Patients diagnosed with AS and CAD did not demonstrate a statistically significant increase in the risk of bleeding complications. It suggests that the use of aspirin does not result in a significant increase in the risk of bleeding and can be utilized in this group.

This study has several limitations. The study was conducted on a relatively small group of participants. Comorbidities in both groups may influence the biomarkers of platelet and endothelial function. Especially that smoking was more prevalent in the CAD group. Thus, the PF4 and TM serum levels might be more elevated secondary to smoking in this group. Regardless of this fact, the PF4 and TM levels were higher in patients with AS. Therefore, it can be concluded that AS had a considerable impact on these findings.

In summary, our findings emphasize the multifaceted role of platelet–endothelial interactions in AS. While structural valvular degeneration has traditionally been the primary focus of AS research, our data suggest that systemic vascular and hemostatic alterations also play a significant role in disease progression and prognosis. The identification of elevated TM and PF4 levels as potential biomarkers of subclinical vascular injury could have prognostic and therapeutic implications.

Future studies with larger cohorts and longitudinal biomarker assessments are warranted to validate these findings and explore their utility in risk stratification and personalized treatment. Additionally, investigating alternative antiplatelet or anti-inflammatory regimens may further elucidate the therapeutic potential of targeting platelet–endothelial interactions in AS.

## 4. Materials and Methods

### 4.1. Materials

A total of 78 adult patients with stable CAD therapy participated in this study (43 males, average age: 74.23 ± 10.2 years), including 49 consecutive patients with AS (27 males, average age: 74.76 ± 12.4 years) and 29 control subjects (16 males, average age: 73.34 ± 4.5 years).

The participants of this study were patients admitted to a local cardiology center between July 2021 and December 2023. The study group consisted of patients with moderate to severe AS who were using aspirin (75 mg per day) as part of their pharmacotherapy. The control group comprised individuals matched for age and gender who did not suffer from aortic valve disease and primarily exhibited CAD, with aspirin (75 mg per day) administered as well.

Both groups underwent comprehensive screening, with a review of their medical history. The following exclusion factors from the study were identified: congenital heart disease, cancer, autoimmune disorders, infections, pregnancy, hematologic conditions (such as thrombophilia or hemophilia A/B), infective endocarditis, chronic kidney disease (stage IV–V, eGFR < 30 mL/min), chronic dialysis, liver dysfunction (elevated hepatic aminotransferases), or those who did not provide informed consent. The baseline characteristics of both groups are summarized in Table 1.

The study was conducted in accordance with the Declaration of Helsinki and approved by the Ethics Committee of Medical University of Silesia, Katowice, Poland (PCN/CBN/0022/KB1/50/21, 6 July 2021). All participants provided written consent before enrollment.

### 4.2. Clinical Assessment

Each participant enrolled in the study underwent a comprehensive clinical evaluation that included a detailed classification according to the New York Heart Association (NYHA) and Canadian Cardiovascular Society (CCS) functional scales. A comprehensive medical history was also obtained, with particular attention to the presence of comorbid conditions, including atrial fibrillation, hypertension, coronary artery disease, diabetes mellitus, dyslipidemia, hypothyroidism, tobacco use, peripheral artery disease, and chronic obstructive pulmonary disease. Standardized physical examinations were conducted, incorporating measurements of anthropometric and hemodynamic parameters such as body weight, height, body mass index (BMI), systolic and diastolic blood pressure, and heart rate. Furthermore, each patient underwent a 12-lead electrocardiogram (ECG), transthoracic two-dimensional echocardiography.

In addition, both study groups were subjected to routine laboratory analyses as part of the diagnostic protocol. The evaluations encompassed a comprehensive array of blood count parameters, including red and white blood cells, hemoglobin, hematocrit, mean corpuscular volume (MCV), neutrophils, lymphocytes, platelet count, mean platelet volume (MPV), and platelet distribution width (PDW). Liver function was assessed through the measurement of alanine aminotransferase (ALT), and metabolic indicators such as glucose were also evaluated. The following variables were measured: creatinine, estimated glomerular filtration rate (eGFR) calculated using the Cockcroft–Gault equation, electrolyte levels (sodium and potassium), thyroid-stimulating hormone (TSH), and lipid profile components such as triglycerides, total cholesterol, low-density lipoprotein (LDL), and high-density lipoprotein (HDL).

### 4.3. Transthoracic Echocardiography

Transthoracic echocardiographic evaluations were performed using a 2.5 MHz transducer and included two-dimensional (2D), M-mode, and Doppler imaging modalities. The echocardiographic assessments were conducted by a cardiologist with extensive experience in the field, in accordance with the guidelines established by the European Association of Cardiovascular Imaging (EACVI) [42]. Furthermore, offline analysis employing two-dimensional speckle-tracking echocardiography was utilized to ascertain left ventricular global longitudinal strain (LV GLS). The echocardiographic protocol encompassed the measurement of several key structural and functional cardiac parameters, including: The following variables were measured: the aortic valve area (AVA), the peak transvalvular velocity (Vmax), the peak (Pmax), and the mean (Pmean) pressure gradients; the left ventricular end-diastolic and end-systolic diameters (LV EDD, LV ESD); the end-diastolic and end-systolic volumes (LV EDV, LV ESV); the left ventricular outflow tract (LVOT) diameter; the left ventricular ejection fraction (LVEF); the stroke volume index (SVi); and the left atrial area (LA area). Furthermore, myocardial wall thicknesses of the interventricular septum (IVS) and the posterior wall (PW) were recorded. Diastolic function was assessed by measuring early mitral inflow velocity (E wave), early diastolic mitral annular velocity (E′), and the E/E′ ratio. Furthermore, the valvulo-arterial impedance (Zva) was calculated.

### 4.4. Biomarkers—Fluorescent Bead-Based Luminex Assays

Venous blood samples (10 mL) were obtained from the antecubital vein of all study participants. Serum specimens designated for analysis were aliquoted into polypropylene tubes and stored at −80 °C until further processing. The quantification of biomarkers was conducted using a magnetic Luminex assay, with the procedure performed in accordance with the manufacturer’s guidelines. Prior to analysis, serum samples were diluted as specified by the protocol and immediately subjected to assay procedures. The concentrations of TM, P-selectin, PF4, and CD40L were determined using multiplex bead-based immunoassays (Luminex technology) on the Bio-Plex 200 suspension array system (R&D Systems, Minneapolis, USA), strictly following the manufacturer’s instructions. The acquisition of data was executed through the utilization of a Bio-Plex 200 instrument, a device that has been validated and calibrated (Bio-Rad Laboratories, Watford, UK). The subsequent processing of results was facilitated by employing Bio-Plex Manager 6.0 software, which is also manufactured by Bio-Rad Laboratories (Watford, UK). For the purpose of assay calibration, the detection target was configured to capture 50 beads per analyte region. This was achieved by employing a low RP1 target for CAL2 calibration. The doublet discrimination gates were set between 5000 and 25,000, in accordance with the recommended settings for the Bio-Plex platform. The primary readout metric—median fluorescence intensity (MFI)—was recorded and utilized for subsequent quantitative analysis.

### 4.5. Platelet Function Tests—Optical Aggregometry

The platelet reactivity following aspirin treatment was measured using the VerifyNow Aspirin assay, while the efficacy of P2Y12 was measured using the VerifyNow-P2Y12 assay. Tests were obtained following the previously published method [43,44]. Blood samples were obtained from all patients and collected in 3.2% citrate Vacuette tubes (Greiner Bio-One, Kremsmünster, Austria). The blood samples were stored at ambient temperature. Tests were carried out in strict accordance with the manufacturers’ guidelines. The test is based on turbidimetric detection, where the extent of light transmittance is proportional to the degree of platelet-induced aggregation of fibrinogen-coated beads. In the VerifyNow Aspirin Assay test (ASPI test), arachidonic acid functions as the platelet agonist. Under normal physiological conditions, arachidonic acid is converted via the cyclooxygenase-1 (COX-1) enzyme pathway into thromboxane A2 (TxA2), a potent stimulator of platelet aggregation. Aspirin exerts its antiplatelet effects by irreversibly inhibiting COX-1, thereby reducing the formation of thromboxane A2 and ultimately preventing the activation of the GPIIb/IIIa receptor, which is essential for platelet aggregation. An aspirin reaction unit (ARU) value of 550 or higher is indicative of normal platelet function and the absence of significant aspirin-induced inhibition, suggesting that the patient is either a non-aspirin user or exhibits aspirin resistance. Conversely, an ARU value below 550 indicates platelet dysfunction, consistent with the presence and efficacy of aspirin. The VerifyNow-P2Y12 assay (ADP test) is a rapid diagnostic test that employs adenosine diphosphate (ADP) to induce platelet activation in the presence of prostaglandin E1 (PGE1), which serves to suppress signaling via the secondary ADP receptor, P2Y12. This design enhances the assay’s sensitivity and specificity for detecting the functional activity of the P2Y12 receptor and the pharmacodynamic effects of P2Y12 receptor antagonists. A result that exceeds 180 P2Y12 reaction units (PRUs) indicates the absence of P2Y12 inhibitor activity, whereas a PRU value of 180 or below reflects a reduced platelet reactivity consistent with the presence of a P2Y12 receptor inhibitor.

### 4.6. A 12-Month Follow-Up

Participants were monitored over a 12-month period through scheduled outpatient clinic visits or, in selected cases, via telephone consultations conducted in the outpatient setting. During the follow-up period, the occurrence of major bleeding (BARC > 1) was systematically documented.

### 4.7. Statistical Analysis

All statistical analyses were conducted using Statistica software, version 13.3 (StatSoft, Krakow, Poland). Continuous variables were expressed as means ± standard deviation (SD) for normally distributed data, or as medians with interquartile ranges (1st and 3rd quartiles) for non-normally distributed data. Categorical variables were presented as absolute counts and corresponding percentages. The Shapiro–Wilk test was used to assess the normality of data distribution. For comparisons between groups, Student’s *t*-test was used for normally distributed continuous variables, while the U Mann–Whitney test was applied to those not meeting normality assumptions. Differences in categorical variables were assessed using the chi-squared test. Person’s coefficient was used to evaluate associations between biomarker concentrations and other continuous or ordinal variables. Variables exhibiting a *p*-value < 0.1 in univariate regression analysis were entered into the multivariate regression model. A two-sided *p*-value < 0.05 was considered indicative of statistical significance.

## 5. Conclusions

This study examined the relationship between AS, endothelial dysfunction, and platelet activation by assessing specific serum biomarkers and platelet aggregation tests. We found that the coexistence of AS in patients with CAD was associated with elevated levels of the aforementioned biomarkers, which are indicative of endothelial damage and platelet activation. On the other hand, the aggregation test representing the efficacy of antiplatelet treatment was comparable between the groups, and no differences in bleeding complications were observed. The findings highlight the role of endothelial and platelet interactions in patients with CAD and AS and suggest the safety of the antiplatelet therapy in this patient population.

## Figures and Tables

**Table 1:** Baseline clinical characteristics. AS—aortic stenosis; CAD—coronary artery disease; BMI—body mass index; NYHA—New York Heart Association; CCS—Canadian Cardiovascular Society; CAD—coronary artery disease; COPD—chronic obstructive pulmonary disease; PAD—peripheral artery disease.

	AS and CAD (N = 49)	CAD (N = 29)	*p* Value
**Male (%), Female%**	27 (55.2%), 22 (44.8%)	16 (55.1%), 13 (44.9%)	0.995
**Age (mean; SD)**	74.76 ± 12.4	73.34 ± 4.5	0.473
**Height (cm)**	166.49 ± 9.3	166.24 ± 17.4	0.668
**BMI (kg/m^2^)**	28.12 ± 4.3	28.04 ± 3.2	0.840
**NYHA class (mean; SD)**	2.51 ± 0.8	1.59 ± 1.1	0.001
**CCS class (mean; SD)**	1.35 ± 1.2	1.72 ± 1.4	0.224
**Hypertension (%)**	42 (91.3%)	27 (93.1%)	0.780
**CAD (%)**	35 (71.4%)	21 (72.4%)	0.926
**Diabetes (%)**	13 (27.1%)	8 (27.6%)	0.962
**Dyslipidemia (%)**	41 (87.2%)	28 (96.6%)	0.172
**Hypothyroidism (%)**	9 (19.1%)	3 (10.3%)	0.307
**Smoking (%)**	7 (14.9%)	12 (41.4%)	0.010
**COPD (%)**	3 (6.5%)	3 (10.3%)	0.552
**PAD (%)**	14 (29.8%)	5 (17.2%)	0.220

**Table 2:** Laboratory characteristics. AS—aortic stenosis; CAD—coronary artery disease; MPV—mean platelet volume; PDW—platelet distribution width; INR—international normalized ratio; APTT—activated partial thromboplastin time; eGFR—estimated glomerular filtration rate; TSH—thyroid-stimulating hormone; LDL—low-density lipoprotein; HDL—high-density lipoprotein.

	AS and CAD (N = 49)	CAD (N = 29)	*p* Value
**Red blood cell [10^6^/μL]**	4.32 ± 0.6	4.43 ± 0.5	0.374
**Hemoglobin [g/dL)**	13.17 ± 1.8	13.32 ± 1.8	0.432
**Hematocrit [%]**	38.77 ± 5	40 ± 4	0.243
**MCV [fL]**	87.9 ± 13.3	90.35 ± 3.9	0.294
**White blood cell [10^3^/μL]**	7.72 ± 1.9	7.11 ± 1.6	0.117
**Neutrophils [10^3^/μL]**	5.02 ± 1.8	4.55 ± 1.2	0.278
**Lymphocytes [10^3^/μL]**	1.74 ± 0.6	1.81 ± 0.5	0.438
**Platelets [10^3^/μL]**	211.92 ± 61.1	217.93 ± 66	0.616
**MPV [fL]**	10.7 ± 0.8	10.87 ± 1	0.559
**PDW [%]**	12.56 ± 1.9	13.01 ± 2.3	0.522
**INR**	1.05 ± 0.1	1.05 ± 0.1	0.923
**APPT [s]**	29.92 ± 4.9	30.05 ± 8.5	0.661
**Glucose [mg/dL]**	103.71 ± 23.9	103.48 ± 15.1	0.678
**Creatinine [mg/dL]**	1.13 ± 0.6	0.9 ± 0.1	0.012
**eGFR Cockcroft-Gault [mL/min]**	64.91 ± 22.2	75.21 ± 15.3	0.009
**Total cholesterol [mg/dL]**	159.85 ± 40.3	144.41 ± 43.7	0.062
**LDL [mg/dL]**	84.48 ± 35.6	74.93 ± 39.5	0.203
**HDL [mg/dL]**	54.54 ± 19.1	49.1 ± 10.7	0.377
**Triglycerides [mg/dL]**	104.58 ± 29.8	101.93 ± 48.4	0.147

**Table 3:** Echocardiography characteristics. AS—aortic stenosis; CAD—coronary artery disease; LV—left ventricular; EDD—end-diastolic diameter; ESD—end-systolic diameter; EDV—end-diastolic volume; ESV—end-systolic volume; EF—ejection fraction; SVi—stroke volume index; GLS—global longitudinal strain; IVS—interventricular septum thickness; PW—posterior wall thickness; Zva—valvulo-arterial impedance; LA area—left atrium area; E/E′—ratio of early mitral inflow velocity to early diastolic mitral annular velocity.

	AS and CAD (N = 49)	CAD (N = 29)	*p* Value
**Left ventricular parameters**			
**LV EDD [mm]**	48.2 ± 6.4	50.62 ± 7.4	0.184
**LV ESD [mm]**	30.41 ± 7.6	30.83 ± 7.2	0.642
**IVS [mm]**	14.49 ± 2.7	11.66 ± 2.9	0.001
**PW [mm]**	11.29 ± 1.6	9.34 ± 1.5	0.001
**LV EDV [mL]**	117.76 ± 40.9	114.76 ± 29.4	0.873
**LV ESV [mL]**	55.25 ± 36.6	51.38 ± 17	0.465
**LV EF [%]**	55.1 ± 11.5	55.17 ± 5.2	0.222
**LV SVi [mL/m^2^]**	36.31 ± 10.5	32.98 ± 6.9	0.149
**LV GLS [%]**	−13.99 ± 3.1	−16.91 ± 3.3	0.001
**Other parameters**			
**Zva [mmHg/mL/m^2^]**	5.5 ± 1.8	4.47 ± 1.1	0.003
**LA area [cm^2^]**	22.41 ± 3.9	21.93 ± 5	0.608
**E/E′**	15.88 ± 6.6	10.43 ± 4.3	0.001

**Table 4:** Results of biomarkers and platelet aggregometry. AS—aortic stenosis; CAD—coronary artery disease; TM—thrombomodulin; PF4—platelet factor 4; ASPI—VerifyNow Aspirin test; ARU—aspirin reaction unit; ADP—VerifyNow P2Y12 test; PRUs—P2Y12 Reaction Units; Q1—first quartile; Q3—third quartile.

	AS and CAD (N = 49)	CAD (N = 29)	*p* Value
**TM [ng/mL]**	7.64 ± 3.5	6.28 ± 2.1	0.011
**PF4 [μg/mL]**	25.16 Q1: 8.3; Q3: 29.6	12.85 Q1: 5.7; Q3: 14.5	0.021
**P-selectin [ng/mL]**	55.83 ± 20.4	54.87 ± 23	0.747
**CD40L [ng/mL]**	4.59 Q1: 2.2; Q3: 6	4.85 Q1: 1.8; Q3: 8.2	0.656
**ASPI [ARU]**	474.04 ± 66.7	471.31 ± 56.2	0.822
**ADP [PRU]**	224.88 ± 46.4	216.62 ± 29.6	0.394

**Table 5:** Pearson’s correlation analysis. TM—thrombomodulin; PF4—platelet factor 4; PRUs—P2Y12 reaction units; r—correlation coefficient; N.S.—not significant.

	Hematocrit	Hemoglobin	CD40L
**P-selectin**	r = 0.2659	r = 0.2471	r = 0.3617
	*p* = 0.019	*p* = 0.030	*p* = 0.011
**PF 4**	N.S.	N.S.	N.S.
**TM**	r = −0.3369	r = −0.2978	N.S.
	*p* = 0.003	*p* = 0.008	
**PRU**	r = −0.4871	r = −0.3797	N.S.
	*p* = 0.001	*p* = 0.001	

## Data Availability

The data presented in this study are available on request from the corresponding author due to privacy, intellectual property rights, and ethical reasons.

## Supplementary Materials

The following supporting information can be downloaded at: https://www.mdpi.com/article/10.3390/ijms26157083/s1.

## Abbreviations

The following abbreviations are used in this manuscript:

AS	aortic stenosis
AVA	aortic valve area
BMI	body mass index
CAD	coronary artery disease
CCS	Canadian Cardiovascular Society
CD	cluster of differentiation
COPD	chronic obstructive pulmonary disease
E/E′	ratio of early mitral inflow velocity to early diastolic mitral annular velocity
EDD	end-diastolic diameter
EDV	end-diastolic volume
EF	ejection fraction
ESD	end-systolic diameter
ESV	end-systolic volume
eGFR	estimated glomerular filtration rate
GLS	global longitudinal strain
HDL	high-density lipoprotein
IVS	interventricular septum thickness
LA area	left atrium area
LDL	low-density lipoprotein
LV	left ventricular
MACCE	major adverse cardiovascular and cerebrovascular event
MCV	mean corpuscular volume
MPV	mean platelet volume
NYHA	New York Heart Association
PAD	peripheral artery disease
PDW	platelet distribution width
PF4	platelet factor 4
Pmax	maximum pressure gradient
Pmean	mean pressure gradient
PW	posterior wall thickness
Q1	first quartile
Q3	third quartile
Svi	stroke volume index
TM	thrombomodulin
Vmax	maximum velocity
VICs	valvular interstitial cells
Zva	valvulo-arterial impedance
