# Supplementary material for: Platelet-Related Biomarkers and Efficacy of Antiplatelet Therapy in Patients with Aortic Stenosis and Coronary Artery Disease
Source: Int J Mol Sci. 2025 Jul 23;26(15):7083. doi: 10.3390/ijms26157083 (PMC12346522; doi:10.3390/ijms26157083)
Supplement: Supplementary file 1 [file ijms-26-07083-s001.zip › ijms-3735559-supplementary.pdf]

	ADP	ASPI	P-selectin	TM	PF4	CD40L	AVA	Vmax	Pmean	Zva	LVEF	E/E'	GLS	Hgb	Hct	RBC	PLT	MPV	PDW	Lymphocytes
**ADP**				0,2522<br>p=0.027					0,2851<br>p=0.012					-0,3797<br>p=0.001	-0,4871<br>p=0.001	-0,4471<br>p=0.001				
**ASPI**																				
**P-selectin**				0,2487<br>p=0.029		0,3617<br>p=0.011								0,2471<br>p=0.030	0,2659<br>p=0.019	0,2617<br>p=0.021	0,2555<br>p=0.025			0,2394<br>p=0.039
**TM**	0,2522<br>p=0.027		0,2487<br>p=0.029				-0,2361<br>p=0.037	0,2332<br>p=0.040						-0,2978<br>p=0.008	-0,3369<br>p=0.003	-0,3319<br>p=0.003				
**PF4**							-0,2614<br>p=0.027	0,2518<br>p=0.033	0,2604<br>p=0.027								0,2683<br>p=0.023			
**CD40L**			0,3617<br>p=0.011																	
**AVA**				-0,2361<br>p=0.037	-0,2614<br>p=0.027			-0,9352<br>p=0.001	-0,8631<br>p=0.001	-0,3718<br>p=0.001		-0,4729<br>p=0.001	-0,4595<br>p=0.001		0,2248<br>p=0.048					
**Vmax**				0,2332<br>p=0.040	0,2518<br>p=0.033		-0,9352<br>p=0.001		0,9687<br>p=0.001	0,3653<br>p=0.001		0,4822<br>p=0.001	0,4178<br>p=0.001		-0,2475<br>p=0.029					
**Pmean**	0,2851<br>p=0.012				0,2604<br>p=0.027		-0,8631<br>p=0.001	0,9687<br>p=0.001		0,336<br>p=0.003		0,4579<br>p=0.001	0,3897<br>p=0.001		-0,2385<br>p=0.035					
**Zva**							-0,3718<br>p=0.001	0,3653<br>p=0.001	0,336<br>p=0.003		-0,2734<br>p=0.015	0,3074<br>p=0.010	0,3469<br>p=0.002							
**LVEF**										-0,2734<br>p=0.015			-0,603<br>p=0.000							
**E/E'**							-0,4729<br>p=0.001	0,4822<br>p=0.001	0,4579<br>p=0.001	0,3074<br>p=0.010			0,3706<br>p=0.002							
**GLS**							-0,4595<br>p=0.001	0,4178<br>p=0.001	0,3897<br>p=0.001	0,3469<br>p=0.002	-0,603<br>p=0.000	0,3706<br>p=0.002								
**Hgb**	-0,3797<br>p=0.001		0,2471<br>p=0.030	-0,2978<br>p=0.008											0,8921<br>p=0.001	0,8343<br>p=0.001	-0,2919<br>p=0.010			
**Hct**	-0,4871<br>p=0.001		0,2659<br>p=0.019	-0,3369<br>p=0.003			0,2248<br>p=0.048	-0,2475<br>p=0.029	-0,2385<br>p=0.035					0,8921<br>p=0.001		0,9364<br>p=0.001				
**RBC**	-0,4471<br>p=0.001		0,2617<br>p=0.021	-0,3319<br>p=0.003										0,8343<br>p=0.001	0,9364<br>p=0.001					
**PLT**			0,2555<br>p=0.025		0,2683<br>p=0.023									-0,2919<br>p=0.010				-0,4595<br>p=0.001	-0,4658<br>p=0.001	
**MPV**																	-0,4595<br>p=0.001		0,9419<br>p=0.001	
**PDW**																	-0,4658<br>p=0.001	0,9419<br>p=0.001		
**Lymphocytes**			0,2394<br>p=0.039																	

Table S1. Pearson's correlation analysis. The table presents the findings of the correlation analysis. The numerical value indicates Pearson's correlation coefficient, and the p-value is displayed below. The table displays only those correlation coefficients that have been statistically significant at the 0.05 level. ADP – P2Y12 test; ASPI –aspirin test; TM – Thrombomodulin; PF4 – Platelet Factor 4; CD40L – CD40 Ligand; AVA – Aortic Valve Area; Vmax – Maximum transvalvular velocity; Pmean – Mean transvalvular pressure gradient; LVEF – Left Ventricular Ejection Fraction;

E/E′ – Ratio of early mitral inflow velocity to mitral annular early diastolic velocity; GLS – Global Longitudinal Strain; Hgb – Hemoglobin concentration; Hct – Hematocrit; RBC – Red Blood Cell count; PLT – Platelet count; MPV – Mean Platelet Volume; PDW – Platelet Distribution Width.
